# A single-source photon source model of a linear accelerator for Monte Carlo dose calculation

**DOI:** 10.1371/journal.pone.0183486

**Published:** 2017-09-08

**Authors:** Obioma Nwankwo, Gerhard Glatting, Frederik Wenz, Jens Fleckenstein

**Affiliations:** 1 Department of Radiation Oncology, Medical Faculty Mannheim, Heidelberg University, Mannheim, Germany; 2 Medical Radiation Physics/Radiation Protection, Medical Faculty Mannheim, Heidelberg University, Mannheim, Germany; 3 Medical Radiation Physics, Department of Nuclear Medicine, Ulm University, Ulm, Germany; North Shore Long Island Jewish Health System, UNITED STATES

## Abstract

**Purpose:**

To introduce a new method of deriving a virtual source model (VSM) of a linear accelerator photon beam from a phase space file (PSF) for Monte Carlo (MC) dose calculation.

**Materials and methods:**

A PSF of a 6 MV photon beam was generated by simulating the interactions of primary electrons with the relevant geometries of a Synergy linear accelerator (Elekta AB, Stockholm, Sweden) and recording the particles that reach a plane 16 cm downstream the electron source. Probability distribution functions (PDFs) for particle positions and energies were derived from the analysis of the PSF. These PDFs were implemented in the VSM using inverse transform sampling. To model particle directions, the phase space plane was divided into a regular square grid. Each element of the grid corresponds to an area of 1 mm^2^ in the phase space plane. The average direction cosines, Pearson correlation coefficient (PCC) between photon energies and their direction cosines, as well as the PCC between the direction cosines were calculated for each grid element. Weighted polynomial surfaces were then fitted to these 2D data. The weights are used to correct for heteroscedasticity across the phase space bins. The directions of the particles created by the VSM were calculated from these fitted functions. The VSM was validated against the PSF by comparing the doses calculated by the two methods for different square field sizes. The comparisons were performed with profile and gamma analyses.

**Results:**

The doses calculated with the PSF and VSM agree to within 3% /1 mm (>95% pixel pass rate) for the evaluated fields.

**Conclusion:**

A new method of deriving a virtual photon source model of a linear accelerator from a PSF file for MC dose calculation was developed. Validation results show that the doses calculated with the VSM and the PSF agree to within 3% /1 mm.

## Introduction

Monte Carlo (MC) dose calculation is considered as the most accurate method of estimating the energy deposited to a medium by ionizing radiation [1]. MC dose calculation in radiation therapy typically starts with a phase space file (PSF) [2–5] or a virtual source model (VSM) [2, 6–14] because it is inefficient to repeatedly simulate particle interactions in the static geometries of the linear accelerator [2, 3, 11, 15]. A PSF is derived by simulating the interactions of primary electrons with the relevant geometries of the modeled device and then recording the particles that reach a reference surface downstream the primary particle source. PSFs contain accurate information about the particles reaching the phase space plane [8] and may be used directly for MC dose calculation as employed in previous works [5, 16]. Due to the large number of particles required for MC dose calculation, PSFs used for dose calculation typically contain several million particles which requires several gigabytes of storage space [13].

MC source models on the other hand contain instructions to create (any desired number of) particles whose properties are a good approximation of those generated by the modeled device or of the information stored in a PSF. They are therefore not limited by the latent variance inherent in PSFs due to their finite size and particle recycling, requires only a few kilobytes or storage space and are the most efficient method of generating particles for MC dose calculation [2, 10, 11, 17, 18]. MC source models may be derived by analytical methods from experimental data [6, 12] or from PSF [8, 11, 13, 19]. A limitation of analytical modeling approaches that use experimental data is that the relevant information for the source model (e.g. energy spectrum) may be difficult to obtain and the information derived by these methods are only a rough approximation of the original data [8]. PSFs on the other hand contain accurate information about the properties of the particles and are therefore a good basis for deriving VSMs. PSF-derived source models have been used extensively for modeling megavoltage photon beams [7–9, 15, 19].

A PSF of a 6 MV Synergy linear accelerator (Elekta AB, Stockholm, Sweden) photon beam was generated and validated in a previous work [5]. Here, we introduce a method to derive a VSM from this reference PSF. Unlike other PSF-based modeling approaches, our generalized source-modeling method neither requires information about the jaws of the linear accelerator [9, 15, 20] nor information/assumption as to the pre-phase space origin of particles [7–9, 13, 20]. In addition, it is based on a single source in contrast to commonly used multiple-source approach [9, 13]. Unique to our method is the approach to modeling particle directions. Firstly, the phase space plane was divided into a grid of square elements. Thereafter, direction information (average direction cosines along the *x*-and *y*-axes and the Pearson correlation coefficient (PCC) between photon energy and direction as well as between the direction cosines) were calculated for the grid elements. Weighted polynomial surfaces fitted to these data constitute our model of particle directions in the VSM. The positions of particles created by the VSM are the input to retrieve their directions from these fitted functions. As our source-modeling method does not require pre- or post-phase space geometric information, it is general in that it can be applied to modeling arbitrarily shaped 2D or 3D surfaces, for example the INTRABEAM device [11].

## Materials and methods

### 2.1 Simulation toolkit, geometric model of linear accelerator and coordinate system

**Fig 1:**
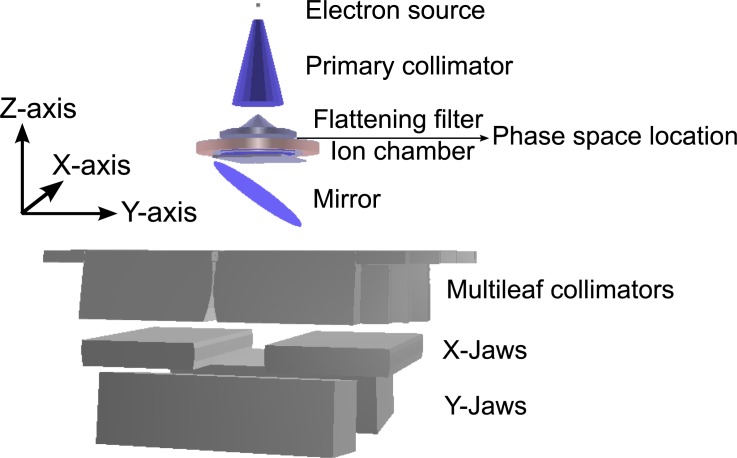
Geometric model of an Elekta Synergy linear accelerator and the coordinate system.

This work was implemented with the Geant4 toolkit [21] (version 4.9.4) for simulating particle transport through matter. The detailed geometry of a linear accelerator was modeled according to available geometric information as shown in Fig 1. The *z*-axis of the coordinate system is in the direction of the primary beam while the *x-y* plane is perpendicular to the beam axis as shown in the figure.

### 2.2 Phase space file (PSF)

A PSF of the 6 MV photon beam was derived from the detailed simulation of the interactions of primary electrons and their secondary products with the relevant geometries of a linear accelerator and recording the particles that reach a scoring plane. The scoring plane is located in the beam axis at a distance of 16 cm from the primary electron source. The 6 particle variables recorded in the PSF are the position in the *x*-*y* plane (*x* and *y*), the direction cosines along the *x*- and *y*-axes (*v*^*x*^ and *v*^*y*^), the energy (*e*), and the particle type (*t*). Additional information regarding the generation and validation of the PSF are available in a previous publication [5]. The PSF used for this work contained 46.12 million particles.

### 2.3 Analysis of the PSF to derive the VSM and particle creation using the VSM

The approach used to derive the VSM from the PSF and the method of creating particles by the VSM will be discussed in the following order

1. Particle position (*x* and *y*)
2. Energy (*e*)
3. Particle direction (*v*^*x*^, *v*^*y*^ and *v*^*z*^**)**
4. Type (*t*)

#### 2.3.1 Particle position (*x* and *y*)

**Analysis of phase space file to derive VSM**

The radial position, *r*_*i*_, of each particle stored in the PSF from the origin of the *x*-*y* plane was calculated,

$$r_{i}=\sqrt{x_{i}^{2}+y_{i}^{2}},$$ (1)

where *i* is the index of the particle in the PSF while *x*_*i*_ and *y*_*i*_ denote its position in the *x-y* plane. The probability density function (PDF) for the radial positions of particles (number of particles per radial distance interval) was calculated using a bin width of 0.1 mm. The cumulative distribution function (*CDF*) was inverted and fitted with a 4^th^ order polynomial spline. The fit to the inverted *CDF*, *F*^−1^, represent the model of particle distribution in the VSM.

**Creating particle position with the VSM**

The radial position, *r*_*i*_, of particles is randomly calculated from *F*^−1^ using the inverse transform sampling method [22] as

$$r_{i}=F^{-1}(U_{1}),$$ (2)

where *U*_1_ is a uniform random number within the closed interval [0, 1]. The location of the particle on the *x-y* plane is calculated from *r*_*i*_ on the basis of rotational symmetry upstream the MLC according to the following equation [11, 15]

$$\left(x_{i},y_{i})=r_{i}(\cos\theta ,\sin\theta \right),$$ (3)

where *θ* is a uniform random angle [0°, 360°], which is obtained by multiplying a uniform random number *U*_*2*_ with 360.

#### 2.3.2 Energy (*e*)

Thirty-five energy spectra were derived from the PSF. Each spectrum represents the energies of particles within a radial distance interval (*h*). The first 25 spectra are for particles the radial distances 0 ≤ *r* ≤ 50 mm (bin width = 2 mm). The next 9 spectra are for the radial distance interval 50 < *r* ≤ 95 mm (bin width = 5 mm), while the last spectrum is for particles with *r* > 95 mm.

The cumulative distribution functions for the energy spectra were inverted and fitted with 5^th^ order polynomial splines (as described in section 2.3.1 for the particle positions). The energy of a particle (*e*_*i*_) was derived from the inverse transform sampling of the corresponding energy spectrum

$$e_{i}=E_{h}^{-1}(U_{3})$$ (4)

where $E_{h}^{-1}$ denotes the inverse cumulative distribution function for the energy spectrum *E*_*h*_ and *U*_3_ denotes a uniform random number.

#### 2.3.3 Particle direction

**Analysis of PSF**

The phase space plane was divided into a regular grid comprising of 141 x 141 elements. Each element of the grid corresponds to an area of 1 x 1 mm^2^ in the phase space. The following variables were then calculated for each grid element, *j*, from the PSF

1. The average direction cosines of the particles along the *x* and *y* axes (${\bar{v}}_{j}^{k}$), calculated as
  $${\bar{v}}_{j}^{k}=\frac{1}{n_{j}}\sum_{i=1}^{n_{j}}v_{i}^{k}$$ (5)
  *k* ∈ {*x*,*y*} and *i* is the index of the particles in the *j*th grid element.
2. The standard error of each average direction cosine ($\sigma_{j}^{k}$), calculated as
  $$\sigma_{j}^{k}=\frac{\sqrt{\frac{1}{n_{j}}\sum_{i=1}^{n_{j}}{\left(v_{i}^{k}-{\bar{v}}_{j}^{k}\right)}^{2}}}{\sqrt{n_{j}}}.$$ (6)
3. The Pearson correlation coefficient [23] (PCC) between the particle energies (*e*) and each lateral (along the *x*- and *y*-axes) direction cosine $\rho_{j}^{eV}$, *V* ∈ {*v*^*x*^,*v*^*y*^}
4. The PCC ($\rho_{j}^{v}$) between the lateral direction cosines (*v*^*x*^ and *v*^*y*^).

Polynomial surfaces of varying degrees were fitted to these 2D data (7 in total). The inverse variance of the average direction cosines at each bin, *j*, was used to weight each data point during fitting as done by other authors [24–26] to correct for heteroscedasticity (inconsistent variance). In other words, each bin element, *j*, was assigned a weight *w*_*j*_ during fitting, where

$$w_{j}^{k}=\frac{1}{\sigma_{j}^{k^{2}}}$$ (7)

This weighting scheme was used for all seven 2D datasets.

**Fits to the average direction cosine data (*v*^*x*^ and *v*^*y*^)**

The phase space was divided into a primary photon region and a secondary (scattered) photon region. Photons in the primary region are defined by the property *r* ≤ *T*, where *r* denotes distance from the origin of the coordinate system and *T* is the distance value considered as the boundary between the two regions. The two regions were determined from the analysis of the PSF. The primary region was considered as all bin elements with particle count equal to or greater than approximately 20% of the number of particles in the bin with the maximum count. A linear and a polynomial surface was then fitted to the primary and secondary regions respectively as expressed in the following equation

$${\hat{v}}^{k}=\{\begin{array}{l} a_{0}^{k}+a_{1}^{k}k+a_{2}^{k}k'\;if\;r\leq T\\ a_{3}^{k}+a_{4}^{k}k+a_{5}^{k}k'+a_{6}^{k}k^{2}+a_{7}^{k}kk'+a_{8}^{k}k^{3}+a_{9}^{k}k^{2}k'\;otherwise \end{array}$$ (8)

*k’* = *y* when *k* = *x* and vice versa, while $a_{i}^{k}$ are the fit coefficients.

**Fits to the standard error of the average direction cosines (*σ*^*k*^) and the PCC data (*ρ*^*eV*^ and *ρ*^*v*^)**

Polynomial surfaces of varying degrees were fitted to the five 2D data, *σ*^*k*^, *ρ*^*eV*^ and *ρ*^*v*^. A single polynomial surface was used to fit both the primary and secondary photon regions (in contrast to the approach used for fitting the data for the average direction cosine). The orders of the polynomial surfaces (in each axis) fitted to *ρ*^*eV*^, *σ*^*k*^ and *ρ*^*v*^ are 3, 4 and 5 respectively. We denote the fits to these data as ${\hat{\rho }}^{eV}$, ${\hat{\sigma }}^{k}$ and ${\hat{\rho }}^{v}$. The order of the polynomial used to fit each data was decided by the visual inspection of the fits and by the adjusted *r*-squared value as suggested by previous authors [27].

**Calculating photon directions in the VSM**

The direction cosine of a photon in the *j*th bin along the *kth*-axis (*x* or *y*) is calculated in the source model as

$$v_{i}^{k}={\bar{v}}_{j}^{k}+\varepsilon_{i}^{k}$$ (9)

where $\varepsilon_{i}^{k}∼N(0,\sigma_{j}^{k})$ is the normally distributed deviation of the direction cosine of the *i*th photon from the bin average, while $v_{i}^{k}$ and $\sigma_{j}^{k}$ are the average direction cosine of the bin and the standard deviation from the mean respectively, which are derived from functions ${\hat{v}}^{k}$ and ${\hat{\sigma }}^{k}$.

A previous study showed that in addition to being dependent on position, photon directions also depends on energy [19]. This correlation is the already mentioned *ρ*^*eV*^. We also considered the correlation, $\rho_{j}^{v}$ between the lateral direction cosines (*v*^*x*^ and *v*^*y*^) in the source model. These correlations (between photon energy and direction and between the lateral direction cosines) were implemented in the source model through $\varepsilon_{i}^{k}$ as described in the following paragraphs.

**Correlating particle energy and direction**

Our technique to incorporating the correlation between energy and direction in the source model is to correlate the uniform random number, *U*_*3*_, that generates photon energies (Eq 4) and the uniform random number *U*_*4*_, that generates the number standard of deviation of the direction cosine of a photon from the bin average. Although the standard deviation of the mean is a normal distribution (hence a normal random number generator is ordinarily expected), a uniform random provides a method to correlate photon energies and their directions. The two uniform random numbers (*U*_*3*_ and *U*_*4*_) were correlated using the sum-of-uniforms (SOU) method [28, 29].

It relevant to briefly describe the SOU method and the approach we used to derive the function relating *parameter c* [28] and the PCC. *Parameter c* is a variable that correlates two independent uniform random variables to a given correlation. Firstly, *c* was specified for the closed interval [0, 10] at a step increment of 0.2. The PCC (between *U*_*3*_ and *U*_*4*_) for each *c* value was determined by generating two independent sequences of 10^6^ random numbers, which were then correlated using *c* according to the SOU method. The PCC between the now correlated random number sequences was then calculated. The function of *c* against PCC was then implemented in the source model and is henceforth referred to as function *g*.

To correlate *U*_*3*_ and *U*_*4*_ in the VSM during simulation, the PCC ($\rho_{j}^{eV}$) between energy and the direction cosine in the *j*th bin is retrieved from the function ${\hat{\rho }}^{eV}$ using the position of the photon as the input to the function. The *c* value that will achieve this correlation ($\rho_{j}^{eV}$) is then determined from function *g* using $\rho_{j}^{eV}$ as the input. This *c* and is then used to correlate *U*_*3*_ and *U*_*4*_. Thereafter, the range of *U*_*4*_ is scaled from [0, 1] to the range [–1, 1] using this [30] technique. The purpose of scaling *U*_*4*_ is to cover the domain of the inverse error function [31] (*erfinv*). The e*rfinv* converts a uniform random number to a normal random number [32], and thus transforms *U*_*4*_ into the normal random number *N*_*U*_. *N*_*U*_ is thus the energy-correlated signed number of standard deviation of the direction cosine of a photon from the bin average.

**Correlating the lateral direction cosines**

It is important to mention at this stage that *U*_*4*_ is generated twice. In other words, two independent random numbers *U*_*4x*_ and *U*_*4y*_ are correlated to *U*_*3*_. Consequently, *N*_*Ux*_ and *N*_*Uy*_ represents number of standard deviations for $\sigma_{j}^{x}$ and $\sigma_{j}^{y}$ respectively. *N*_*Ux*_ and *N*_*Uy*_ are correlated against each other using a previously described technique to correlate normal random numbers. Firstly, the PCC between the two direction cosines in the *j*th bin $\rho_{j}^{v}$ is retrieved from function ${\hat{\rho }}^{v}$. *N*_*Ux*_ and *N*_*Uy*_ are then correlated against each other using a previously described method [33] of correlating normal random numbers

$$N_{Ux}=\rho_{j}^{v}\times N_{Uy}+\left(\sqrt{1-\rho_{j}^{v^{2}}}\right)\times N_{Ux}$$ (10)

Hence, $\varepsilon_{i}^{k}$ which appears in Eq 9 is calculated as

$$\varepsilon_{i}^{k}=N_{Uk}\sigma_{j}^{k}$$ (11)

**Direction cosine along the z-axis (*v*^*z*^)**

The z-axis in the reference co-ordinate system defines the direction of the primary beam illustrated in Fig 1. The direction cosine of each particle along the *z*-axis, $v_{i}^{z}$, was calculated as follows

$$v_{i}^{z}=\sqrt{1-\left(v_{i}^{x^{2}}+v_{i}^{y^{2}}\right)}$$ (12)

#### 2.3.4 Type of radiation (*t*)

The proportion of the different particle types in the PSF was determined. The type of radiation *t*_*i*_ (photon or electron) generated by the VSM was determined according to the following equation

$$t_{i}=\{\begin{array}{c} \gamma \;if\;U_{5}\leq P^{\gamma }\\ e\;otherwise \end{array},$$ (13)

where *U*_5_ is a uniform random number and *P*^*γ*^ is the proportion of photons in the PSF (such that *P*^*γ*^ + *P*^*e*^ = 1).

### 2.4 Summary of the process of creating photons by the VSM

**Fig 2:**
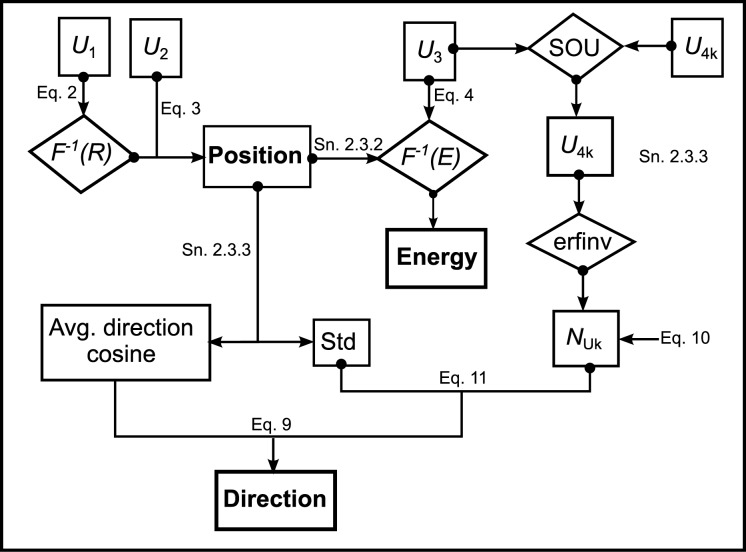
The figure summarizes the process of creating photons in the source model and the interdependency of the photon variables.

Fig 2 provides illustrates the process of creating photons in the source model and the interdependency among the photon variable. Six uniform random numbers are used to generate the photon variables in the following sequence

1. Two uniform random numbers (*U*_*1*_ and *U*_*2*_) are used to generate the position of the photon as described by Eqs 2 and 3.
2. The energy spectrum corresponding to the radial position of the photon is sampled using the uniform random number *U*_*3*_ as described by Eq 4.
3. The direction of the photon is calculated as described in section 2.3.3 and depends on both the position and the energy of the photon. Two uniform random numbers *U*_*4x*_ and *U*_*4y*_ are required for this purpose.
4. The type of radiation (photon or electron) is generated with the uniform random number *U*_*5*_ independent of the other photon variables.

### 2.5 Dose calculation and VSM validation

Two sets of calculations were made using the PSF and the VSM. The doses calculated with the PSF [5] were considered as the reference as our goal is to replace the PSF in the original MC dose calculation algorithm with a VSM. The same dose calculation scheme (voxel size, no variance reduction techniques, dose scoring method) described for the PSF [5] was also used for the VSM. The PSF was used as the particle source to calculate the dose to water for five square field sizes (3 x 3 cm^2^, 5 x 5 cm^2^, 10 x 10 cm^2^, 20 x 20 cm^2^ and 30 x 30 cm^2^). Thereafter, the particle source was changed to VSM and the doses for the same 5 field sizes were recalculated. The doses were then compared using profile and gamma analysis [34, 35]. The gamma analysis was performed with OmniPro I’mRT software (IBA, Dosimetry GmbH, Schwarzenbruck, Germany) using the 3% / 1 mm and 3% / 2 mm analysis criteria. The threshold dose considered for the gamma analysis was 10% of the maximum dose and a pixel pass rate of at least 95% is deemed as the passing criterion.

## Results

### 3.1 Particle position

**Fig 3:**
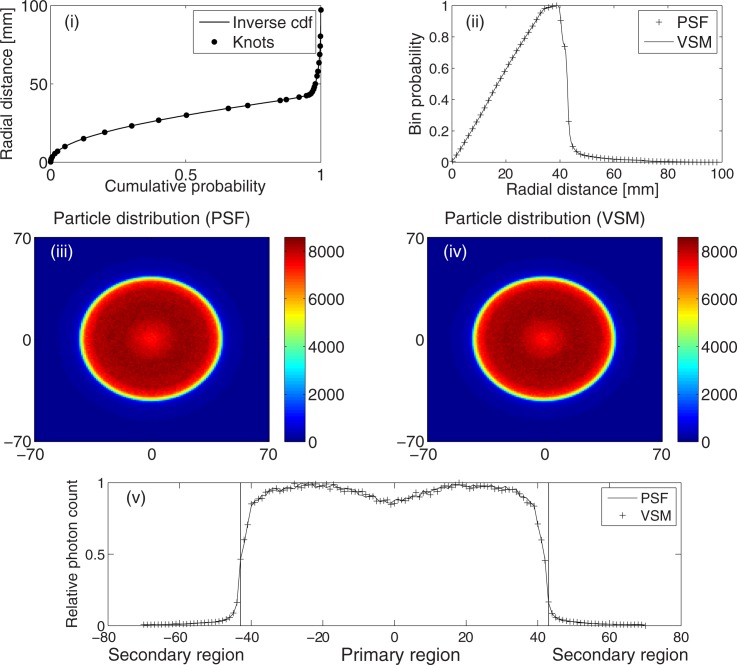
Particle positions. (i) Inverse cumulative distribution function for radial positions of particles (continuous line). The knots show the boundaries of the 30 polynomial segements that make up the fitting spline. (ii) Comparison of the radial positions of the PSF particles and those created by the VSM on the phase space. (iii) Distribution of 46.12 million PSF particles on the x-y plane. (iv) Distribution of 46.12 million VSM particles on the x-y plane. (v) Horizontal profile through the centre of the phase spaces.

Fig 3 shows the distribution of photons in the phase space and our model of particle distribution. Fig 3(i) shows the inverse cumulative distribution function (continuous line) for the radial position of particles. The knots show the boundaries of the 30 segments of the fitting spline. Fig 3(ii) compares the radial positions of the (46.12 million) particles stored in the PSF and those created by the VSM, while Fig 3(iii) and 3(iv) compare their positions on the x-y plane. Fig 3(v) *shows the horizontal profiles through the centre of the PSF and VSM phase spaces, i.e. *profiles through the centre of*Fig 3(iii) and 3(iv). The two vertical lines show the primary and the secondary photon regions as explained in section 2.3.3. Photons in the primary region constitute* 95.62% of all photons in the PSF. The separation of the phase space into two regions was useful for modeling photon directions. The results show that this approach is accurate for modeling particle positions in the source model.

### 3.2. Energy

**Fig 4:**
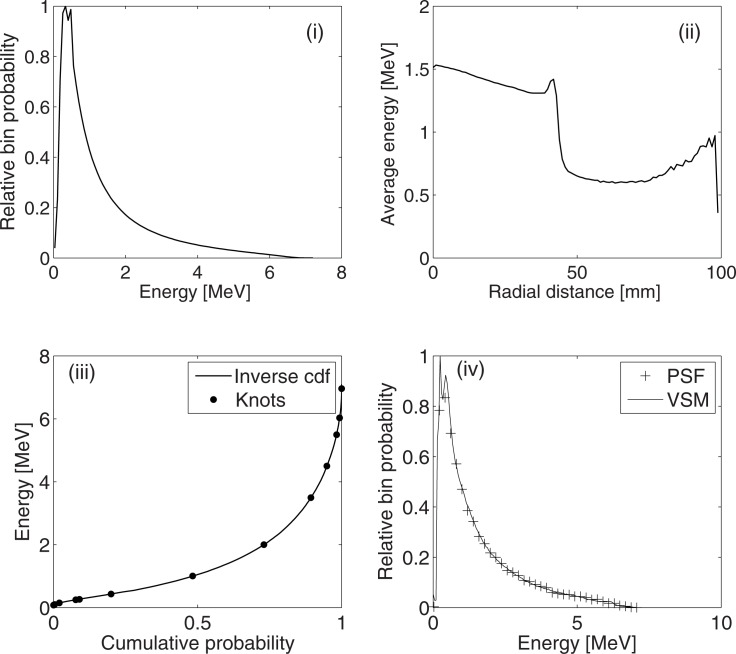
(i) Energy spectrum of all particles in the PSF. (ii) Average energies of photons as a function of distance from the centre of the beam (iii) Fit to the inverse cumulative distribution function for the first energy spectrum (photons with r ≤ 2 mm). The continuous line represents the inverted cumulative distribution function while the knots show the boundaries of the 12 polynomial segments of the fitted spline (iv) Comparison of the energy spectrum of particles stored in the PSF and those created by the VSM for r ≤ 2 mm.

Fig 4(i) shows the energy spectrum of all the particles in the VSM and Fig 4(ii) shows the average photon energies as a function of their distance from the centre. The results show that the average photon energy tends to decrease with increasing distance away from the centre. Fig 4(iv) compares the energy spectrum of the photons in the PSF and those created by the VSM for *r* ≤ 2 mm. The result shows good agreement between the reference (PSF) energy spectrum and the spectrum of the photons created by the VSM. All the energy spectra (for the various distance bins) were visually verified as shown in Fig 4(iv).

### 3.3 Particle directions

**Fig 5:**
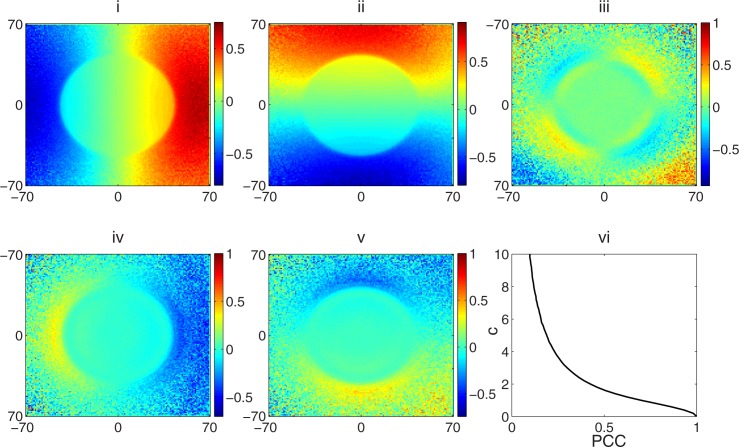
Analysis of the PSF with respect to particle directions and the model of particle directions in the VSM. Average direction cosines along the (i) x-axis and 5 (ii) y-axis. (iii) PCC between the lateral direction cosines. (iv) PCC between photon energies and the direction cosines along the x- and (v) PCC between photon energies and the direction cosines along the y-axis. (vi) Function relating parameter c and the PCC.

Fig 5 shows the result of the analysis of the PSF with respect to photon directions. Fig 5(i) and 5(ii) show the average direction cosines along the *x*- and *y*-axis respectively. The pixel values in the subfigures are the result of averaging the direction cosines of all photons within a phase space area of 1 mm^2^. The threshold distance *T*, which describes the boundary between the primary and secondary photon regions as expressed in Eq 8 was considered to be 43 mm. The *primary* photons (as discussed in section 2.3.3) constitute 95.62% of all photons in the phase space. This boundary region between the primary and secondary photons is shown in Fig 3(v).

Fig 5(iii) shows the PCC between the lateral direction cosines, while Fig 5(iv) and 5(v) show the PCC between photon energies and the direction cosines along the *x*- and *y*-axis respectively. The results show that the divergence (lateral direction cosines) of the beam decreases as the photon energy increases. This inference is based on the fact that the PCC value is positive in the region where the average direction cosines are negative; hence the direction cosines are increasing in the positive direction (however, the absolute value is decreasing) as the photon energy increases. Conversely, the PCC values are negative in the region where the average direction cosines are positive (Fig 5(iv) and 5(v) should thus be analyzed in conjunction with Fig 5(i) and 5(ii)), In other words, the beam divergence decreases as the photon energy increases. Fig 5(vi) shows the function relating PCC and *parameter c*. *Parameter c* is the variable required to correlate two independent uniform random variables with the sum-of-uniforms method. Weighted polynomial surfaces fitted to these 2D data (including the data for the standard error of the average direction cosines which are not shown) as well the function relating *c* and PCC constitute our model of photon directions in the VSM.

### 3.4 Type of radiation

Analysis of the PSF showed that it is composed of 99.3% photons and 0.7% electrons. The type of particle created by the VSM was determined as described in Eq 13.

### 3.5 Comparison of the doses calculated with PSF and VSM

**Fig 6:**
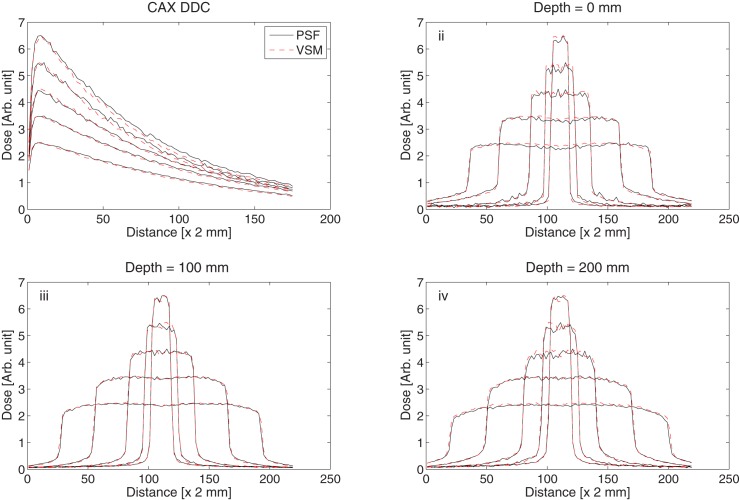
Comparison of the calculated dose with the PSF and VSM algorithms. (i) Central axis depth dose curves. Lateral profiles at (i) 0 mm (ii) 10 cm and (iii) 20 cm depths for field sizes 3 x 3 cm^2^, 5 x 5 cm^2^, 10 x 10 cm^2^, 20 x 20 cm^2^ and 30 x 30 cm^2^.

Fig 6 and Table 1 compare the dose to water calculated using the PSF and VSM as the particle source for 5 field sizes (3x 3 cm^2^, 5 x 5 cm^2^, 10 x 10 cm^2^, 20 x 20 cm^2^ and 30 x 30 cm^2^). Fig 6(i) compares the central axis depth dose curves while Fig 6(ii), 6(iii) and 6(iv) compare the lateral profiles at 0 cm, 10 cm and 20 cm depths respectively. Table 1 shows the results of the comparison of the datasets using the gamma analysis method. All the fields pass with the 3% / 1 mm analysis criterion.

**Table 1:** Comparison of the PSF and VSM-calculated central axis dose planes using gamma analysis.

*Field size (cm*^*2*^*)*	*Gamma 3% / 1 mm*	*Gamma 3% / 2 mm*
**3 x 3**	97.83	99.08
**5 x 5**	97.99	99,13
**10 x 10**	99.35	99.83
**20 x 20**	99.94	100
**30 x 30**	98.42	99,99

## Discussion

A new method of modeling the phase space of a linear accelerator photon beam using a VSM for MC dose calculation is introduced. Unlike previous VSM implementation methods, our source-modeling approach does not require information about the jaws of the linear accelerator [15, 20] nor pre-phase space information/assumption of the origin of particles [7, 8]. The non-requirement of geometric information regarding the construction of the modeled device makes our approach more general and applicable to modeling arbitrarily shaped 2D and 3D phase space surfaces, such as the INTRABEAM system [11]. Additionally, the non-utilization of pre-phase space geometric information implies that given a PSF, the detailed pre-phase space geometric information about the construction of the source (proprietary data) is not required to derive a source model.

VSMs are commonly implemented using multiple sub-sources placed at different geometric locations [9, 12, 13, 36]. Up to 12 sub-sources have been used for modeling a linear accelerator photon beam [9]. The justification for the use of multiple sources is that particles originating from different components of the linear accelerator have different properties (distributions, energy and directions) while those from the same accelerator components possess similar properties [13]. Hence, the multiple source approaches create particles at the different locations of the components of the linear accelerator. But the PSF is scored/defined on a single plane. Hence, our goal is to demonstrate that it is possible to define a VSM that is located at exactly the position of the phase space plane. We have thus introduced a method to reconstruct the PSF using a single particle source.

Unique to our source modeling is the method of modeling particle directions. Particle directions in other source-modeling approaches require pre-phase space information regarding the origin of the photon [7–9, 13, 20]. This information is not required with our approach. Our method is to firstly divide the phase space plane into a regular square grid and then group particles according to their location in the phase space plane. Thereafter, direction information (the average direction cosines and their standard errors, the PCC between photon energy and the direction cosines, as well as the PCC between the lateral direction cosines) are calculated for each grid element. Polynomial surfaces fitted to these data constituted our model of particle directions in the VSM. The grid size used for modeling particle directions corresponds to an area of 1 mm^2^ in the phase space plane. The influence of varying grid size on the accuracy of the VSM was not investigated. Hence, an optimized grid size could yield improved results.

The dose calculated with the PSF was considered as reference in this work because our aim is to demonstrate how to approximate the PSF data with a VSM. The inverse-standard error [24, 37–40] and inverse-variance [24–26] techniques are commonly used weighting schemes to correct for inconsistent variances [38]. We found the inverse-variance approach as suitable weights for fitting the data on photon directions.

It was shown in a previous work [19] that photon directions also depend on their energies (in addition to being position-dependent). We confirmed this correlation in our analysis of the PSF as shown in Fig 5(iv) and 5(v). We also developed a new method to correlate photon energies and their directions by correlating the uniform random numbers that generate photon energies (*U*_*3*_) to the uniform random number (*U*_*4*_) that generates the number of standard deviation of their direction cosines from the bin average. It is relevant to explain how the correlation of these two uniform random numbers will translate to photon directions that are correlated with their energies.

With the inverse transform sampling method that was used to generate the photon energies, small values of *U*_*3*_ (close to 0) generate low energies; intermediate values (around 0.5) generate the average energies in the spectrum, while large values (close to 1) generate high energies. For the inverse error function [31], low values (close to -1) generates large negative number of standard deviation, intermediate values (close to 0) generate values close to 0, while large values (close to 1) generate large positive number of standard deviation.

To explain how the forgoing translates to photon directions that are correlated to their energies, we first consider the instance of positive correlation, which is the case in the phase space region where the average direction cosines are negative (Fig 5(i), 5(ii), 5(iv) and 5(v)). Although the results show a moderate level of correlation between photon energies and directions, let’s assume a perfect correlation (-1 or 1) of these variables for the purpose of this discussion. High photon energies (generated by large *U*_*3*_ values) will lead to the generation of a positive large number of standard deviation due to the positive correlation between *U*_*3*_ and *U*_*4*_. The effect of adding this large positive number of standard deviation to the average direction cosine of the bin (Eq 9) is that the direction cosine of the photon increases in the positive direction (absolute value is decreased). The implication is that the photon becomes less divergent as the energy of the photon increases. The opposite is true when the photon energy is low. In this case, large negative number of standard deviations is generated (due to the low *U*_*3*_ and *U*_*4*_ values), which increases the lateral direction cosine in the negative direction.

In the case of negative correlation, which is the situation in the region where the direction cosines are positive as can be seen in Fig 5(i), 5(ii), 5(iv) and 5(v), high photon energies generate large negative number of standard deviation, which when added to the positive average direction cosine value leads to a decrease in the lateral direction cosine of the photon. Conversely, the divergence (lateral direction cosine) of the photon increases as photon energy decreases because low *U*_*3*_ values (which generates low photon energies) will lead to large *U*_*4*_ values being generated (due to negative correlation), which in turn translates to a large positive number of standard deviation, which increases the lateral direction cosine (divergence) of the photon.

## Conclusion

A method of deriving a virtual photon source model of a linear accelerator from a PSF is introduced. Unique to the approach is the method of handling particle direction, which does not require pre- or post- phase space information. Additionally, the VSM was implemented with a single particle source that is located at the plane where of the PSF was scored. Validation results show a 3% / 1 mm agreement between the doses calculated with the PSF and VSM.

## Supporting information

S1 File Phase space file containing 500,000 particles. The test file is an N x 6 matrix (N = 500,000). The first column specify the type of radiation (|1| = photon, |2| = electron, |3| = positron), the second column the energy (MV), the 3^rd^ and 4^th^ columns the x and y positions (mm), and the last 2 columns the direction cosines along the x- and y-axis.
(ZIP)

S2 File Phase space file containing 500,000 particles. Same format as S1 File.
(ZIP)

S3 File Phase space file containing 500,000 particles. Same format as S1 File.
(ZIP)

S4 File Phase space file containing 500,000 particles. Same format as S1 File.
(ZIP)

S1 Dataset Dataset containing the reference (PSF) central axis dose planes of all field sizes. (ZIP)

S2 Dataset Dataset containing the central axis dose planes calculated using the virtual source model as the particle source. (ZIP)
